# Comparative Performance of Different Traps for Collection of Phlebotominae Sand Flies and Estimation of Biodiversity Indices in Three Endemic Leishmaniasis Foci in North Khorasan Province, Northeast of Iran

**Published:** 2019-12-31

**Authors:** Kourosh Arzamani, Yavar Rassi, Hassan Vatandoost, Amir Ahmad Akhavan, Mohammad Reza Abai, Mohammad Alavinia, Kamran Akbarzadeh, Mehdi Mohebali, Sayena Rafizadeh, Fateh Karimian, Mehdi Badakhshan, Azad Absavaran

**Affiliations:** 1 Vector-Borne Diseases Research Center, North Khorasan University of Medical Sciences, Bojnurd, Iran; 2 Department of Medical Entomology and Vector Control, School of Public Health, Tehran University of Medical Sciences, Tehran, Iran; 3 Department of Environmental Chemical Pollutants and Pesticides, Institute for Environmental Research, Tehran University of Medical Sciences, Tehran, Iran; 4 Toronto Rehabilitation Centre, University Health Network, Toronto, Canada; 5 Department of Medical Parasitology, School of Public Health, Tehran University of Medical Sciences, Tehran, Iran; 6 Ministry of Health and Medical Education, Tehran, Iran

**Keywords:** Collection method, Biodiversity, Sandflies, *Leishmania*, Iran

## Abstract

**Background:**

Phlebotominae sand flies (Diptera: Psychodidae) are the vectors of leishmaniasis. There are different methods for sand fly collection with different performance. The purpose of the current study was to compare the effect of different traps for collection of Phlebotominae sand flies in three endemic leishmaniasis foci in North Khorasan Province, northeast of Iran.

**Methods:**

Sand flies were collected using seven different traps from three villages, three times each twenty days during peak periods of seasonal activity in 2016.

**Results:**

A total of 7253 sand flies were collected. The specimens belonged to19 species. *Phlebotomus sergenti* was the most predominant species in the study area. Light trap baited with Carbon dioxide (CLT) and sticky paper trap (SPT) caught 22.6% and 22.3% of sand flies respectively. Animal baited trap (ABT) and white Shannon trap (WST) caught significantly fewer sand flies than the other traps. The sex ratio was different by phlebotominae sand fly species and collection methods. The sex ratio was highest in SPT and lowest in black Shannon trap (BST). Species diversity and species richness in SPT were more than other traps.

**Conclusion:**

Our findings confirm that CLT and SPT are the most efficient sand fly collection methods. CLT is higher attractive for females and Phlebotomus genus and is an ideal method for monitoring the population of Phlebotomus genus during surveillance. SPT is an inexpensive, convenient and easy to be used to detect the presence of sand flies at low densities and provide a more realistic estimation of sand flies biodiversity.

## Introduction

Leishmaniasis is transmitted to human in 98 countries and 3 territories on 5 continents in the world. There are three main forms of leishmaniases, visceral leishmaniasis (VL), cutaneous leishmaniasis (CL), and mucocutaneous leishmaniasis (1).

In Iran, VL and CL are endemic and this country is one of the ten countries with the highest estimated cases of CL. In the North Khorasan Province VL and CL are endemic. *Leishmania infantum* have been detected in reservoir and vector of the disease. Numerous cases of VL have been recorded during the last decades in humans. About 160 confirmed human cases of VL have been diagnosed and registered especially from the north half of the province during the period 1990–2010. During 2005 to 2008, about 1453 and between the years 2006–2013 at least 2831 patients with CL were reported from different districts of the province (2–7).

Phlebotominae sand flies (Diptera: Psychodidae) are the vectors of leishmaniasis and some human pathogens. There are approximately 1,000 valid described species of sand flies in the world (8).

The first step for study of Phlebotominae sand flies, their behavior, taxonomy, ecology or determination of infection is collection of them. There are several methods and traps for collection of phlebotominae sand flies (9). A variety of phlebotominae sand flies collection methods have previously been studied in different countries. In a study in Turkey among different traps, light trap baited with Carbon dioxide (CLT) were found to be more stable and productive method than others trap for both estimating the species composition and the population density of sand flies in the study area (10).

In an investigation in Morocco statistical analysis showed that significantly more number of sand flies were obtained using sticky paper traps (SPT) compared with light traps (LTP), therefore SPT was more effective than LTP (11).

In northeastern of Italy three standard methods for collection of sand flies (SPT, LTP, and CLT) were compared. CLT were more attractive for females of *P. perniciosus* and *P. neglectus*. LTP showed an intermediate efficiency and were more attractive for *P. neglectus*, compared to other two traps. Results suggest that in northern Italy the CLT is a suitable sampling method for sand fly monitoring programs (12).

In center of Iran eight methods for collection of sand flies were compared and 37.3% of all phlebotominae sand flies were collected using Disney trap (DST). These proportions were 24.2%, 10.5%, 8.5% and7.3% in SPT, Black Shannon Traps (BST), Animal Baited Trap (ABT) and White Shannon Traps (WST) respectively. CLT (5.0%), LTP (3.3%) and Malaise trap (0.1%) showed a low efficiency (13).

In Brazil to compare the relative attractiveness of BST and WST for sand flies, several pairs of traps were placed side by side in front of caves in four areas. The result revealed that BST was much more productive than the WST, especially for anthropophilic species (14).

According to the previous study tolerance to DDT reported in North Khorasan Province, therefore collection and monitoring of phlebotominae sand flies is necessary (15). There is no sufficient document for evaluation of different collection methods of phlebotominae sand flies in Iran. The purpose of this study was to compare performance of different traps in three endemic leishmaniasis foci in North Khorasan Province, northeast of Iran to assess which of these traps could catch a large number or show the most diversity of sand flies.

## Materials and Methods

### Study area

**Fig. 1.**
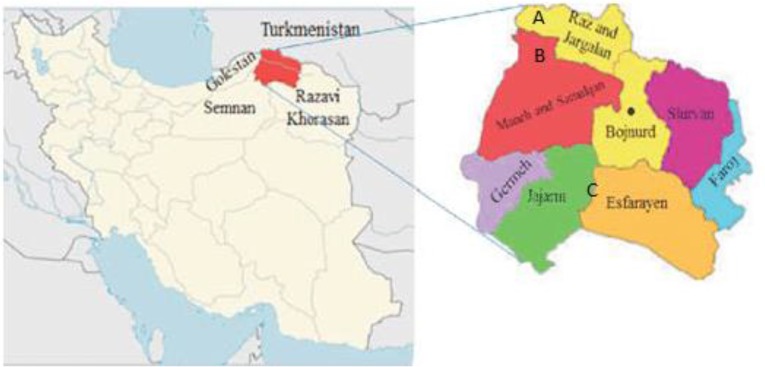
Study area of Phlebotomine sand flies collection, North Khorasan Province, Iran, 2016. A: Bacheh-Dareh, B: Kohne-Jolgeh, C: Arg

This cross-sectional study was conducted during 2016 in North Khorasan Province, between 36°37′-38°17′ N latitudes and 55°53′-58°20′ E longitudes. The province has a desert, mountain and temperate climate with cold winters and receives about 250mm of rainfall annually. The total area was approximately 28,434km^2^. The province is bordered by Turkmenistan in the North and situated in northeast of Iran. Bojnurd is the capital city of the province (Fig. 1).

### Selection of study villages

Three villages in the province were selected, where human VL and/or CL had been reported in the last 5 years. Bacheh-Dareh village (A): It is located in Raz and Jargalan County, 15km from the border of Turkmenistan. Several human VL and CL cases had been reported in the last 5 years. Collection site was a valley 3km far from the village and sand flies captures have been restricted to outdoor and wild environments. Kohne-Jolgeh village (B): It is located in Maneh and Samalqan County. Several human VL and CL had been reported in the last 5 years. Collection site was indoors and domestic environments. Arg village (C): It is located in Jajarm County. Only human CL cases had been reported from this village. Collection site was indoors and outdoors and domestic environments.

### Sand fly collection

**Fig. 2.**
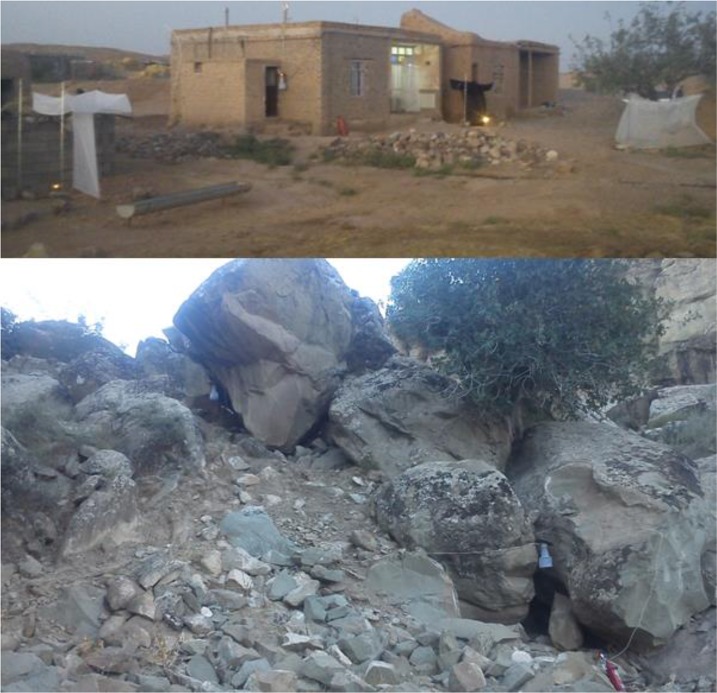
Different collection methods used for sampling of phlebotomine sand flies, North Khorasan Province, Iran, 2016. Above: white and black Shanon and animal baited traps in location C. Below: Light trap and light trap baited with Carbon dioxide gas in location A

Sand flies were collected for three times each twenty days during the peak periods of seasonal activity. Sample collection began from early Jul and continued until late Aug in 2016. Sand flies were collected using seven different traps including:

1. Sticky paper Traps (SPT) consisting of a white paper sheet with size of 15×21cm that coated with castor oil. We used 10 paper traps for each rotation and totally 60 papers per night.
2. CDC Light Trap (LTP). The light traps powered by alternating current and were suspended at 1.5 meters above the ground.
3. CDC light trap baited with Carbon dioxide (Co_2_) gas (CLT) that obtained by a Co_2_ gas tank.
4. White Shannon Trap (WST) was made of white cloth and consisted of a large central compartment and two smaller lateral ones. The measurements, (width, length and height), of the central and the lateral compartments were, respectively: 1.3×1.3×2 meters and suspended by cords from supports and base of the traps touched the ground. A portable stove was used as Co_2_ and light source inside the traps.
5. Black Shannon Trap (BST). This trap was similar to WST except that were made of black cloth.
6. Disney Trap (DST). A cage, holding the chicken bait in the middle and surrounded by sticky paper traps.
7. Animal Baited Trap (ABT). A net trap (2×3×2 meters) was used to sample sand flies attracted to a chicken as animal bait (Fig. 2).

All of the traps were placed randomly with a distance of about 20 meters of each other. The traps were set before sunset, were changed every two hours and remained in operation during the night (20:00 to 08:00). As the traps were changed, the new ones were replaced in the same location. Traps were rotated clockwise between the trap locations in site “A” and “C” but were fix in site “B”. Collected sand flies were stored in 96% ethanol alcohol. The specimens were mounted on glass slides in Puri’s medium. Species identification was carried out according to morphological characters with several taxonomic keys of sand flies (16–17).

### Statistical analyses

The statistical analyses were performed in SPSS program. We evaluated the comparative performance of different traps for each collection site. Shannon-Wiener index have been calculated to estimate species biodiversity of Phlebotomine sand flies in different traps (18–19).

### Ethical statement

Specimen collection was performed in accordance with the procedures approved by the Ethical Committee of North Khorasan University of Medical Sciences.

## Results

A total of 7253 sand flies were collected. Frequency, gender and percent of Phlebotomine sand flies collected by different traps in three collection sites are shown in Table 1. On collection site A we collected the most abundance of sand flies and in collection site C the fewer of specimens collected.

**Table 1.** Frequency, gender and percent of sand flies collected by different traps in three endemic Leishmaniasis Foci, North Khorasan Province, 2016

**Method**	**location**											

	**A**				**B**				**C**			

	**female**	**male**	**total**	**%**	**female**	**male**	**total**	**%**	**female**	**male**	**total**	**%**
**ABT**	59	140	199	6.5	39	68	107	4.5	122	80	202	11.3
**BST**	183	147	330	10.7	80	85	165	6.9	245	45	290	16.2
**CLT**	316	328	644	21.0	401	289	690	28.8	172	133	305	17.1
**DST**	330	373	703	22.9	118	220	338	14.1	101	61	162	9.1
**LTP**	134	137	271	8.8	253	289	542	22.7	71	67	138	7.7
**SPT**	257	581	838	27.3	134	272	406	17.0	226	144	370	20.7
**WST**	30	58	88	2.9	68	76	144	6.0	204	117	321	18.0
**Total**	1309	1764	3073	100.0	1093	1299	2392	100.0	1141	647	1788	100.0

ABT: Animal baited trap; BST: Black Shannon trap; CLT: Light trap baited with Carbon dioxide; DST: Disney trap; LTP: CDC Light Trap; SPT: Sticky paper traps; WST: White Shannon trap. A: Bacheh-Dareh; B: Kohne-Jolgeh; C: Arg

The specimens were belonged to19 species, 10 species of the genus *Phlebotomus* and 9 of the genus *Sergentomyia*. In A collection site, we collected 18 species and more sand fly species collection appears in this area. Significantly fewer sand flies collected in site C with A collection of only four species and in site B eight species were caught. *Phlebotomus sergenti* was the most predominant species being recorded in all localities. Abundance of Phlebotomine sand fly species, collected by different traps is shown in Table 2.

**Table 2.** Species abundance of sand flies collected by different traps, North Khorasan Province, 2016

**Species**	**Collection methods**						

	**ABT**	**BST**	**CLT**	**DST**	**LTP**	**SPT**	**WST**
***Ph.sergenti***	179	421	1090	918	642	869	189
***Ph. papatasi***	238	298	386	179	207	226	319
***Ph. alexandri***	20	2	94	3	23	56	15
***Ph. major***	3	15	11	3	6	23	0
***Ph. halepensis***	5	2	5	2	5	10	1
***Ph. caucasicus***	0	0	1	0	3	4	0
***Ph. mongolensis***	3	0	1	2	1	2	0
***Ph. longiductus***	0	0	0	2	3	0	0
***Ph. turanicus***	0	0	1	1	0	1	0
***Ph. ansarii***	0	0	0	0	0	1	0
***Se. sintoni***	17	18	13	41	11	219	12
***Se. sumbarica***	22	2	22	16	15	53	5
***Se. pawlowskyi***	2	3	0	12	7	48	0
***Se. theodori***	0	0	8	11	5	43	0
***Se. dreyfussi***	8	21	4	7	3	20	11
***Se. hodgsoni***	7	1	3	2	10	17	1
***Se. dentata***	4	0	0	4	8	17	0
***Se. clydei***	0	2	0	0	2	1	0
***Se. grekovi***	0	0	0	0	0	4	0
**Frequency**	508	785	1639	1203	951	1614	553

ABT: Animal baited trap; BST: Black Shannon trap; CLT: Light trap baited with Carbon dioxide; DST: Disney trap; LTP: CDC Light Trap; SPT: Sticky paper traps; WST: White Shannon trap

More than 89.5% of specimens belong to genus *Phlebotomus* and 62.6% of collected specimens belong to subgenus *Paraphlebotomus*. Frequency of sand flies by genus and percent collected by different traps are shown in table 3. CLT (22.6%) and SPT (22.3%) caught significantly more sand flies and ABT (7%) and WST (7.6%) caught significantly fewer sand flies than the other traps. Statistical analysis showed significant difference between collection methods and genus of collected sand flies, χ^2^= 577.8 and p< 0.001.

**Table 3.** Frequency of sand flies by genus collected by different traps, North Khorasan Province, 2016

**CM**	**Genus**				**Total**	**percentage**

	***Phlebotomus***	**percentage**	***Sergentomyia***	**percentage**		
**ABT**	448	6.9	60	7.9	508	7.0
**BST**	738	11.4	47	6.2	785	10.8
**CLT**	1589	24.5	50	6.6	1639	22.6
**DST**	1110	17.1	93	12.2	1203	16.6
**LTP**	890	13.7	61	8.0	951	13.1
**SPT**	1192	18.4	422	55.4	1614	22.3
**WST**	524	8.1	29	3.8	553	7.6
**Total**	6491	100.0	762	100.0	7253	100.0

CM: Collection methods; ABT: Animal baited trap; BST: Black Shannon trap; CLT: Light trap baited with Carbon dioxide; DST: Disney trap; LTP: CDC Light Trap; SPT: Sticky paper traps; WST: White Shannon trap

The sex ratios were different in phlebotominae sand fly species. Sex ratios in *Ph. sergenti*, *Ph. alexandri* and *Ph. papatasi* were 130, 166 and 75 respectively. In *Sergentomyia* genus sex ratio were 21 and 181 in *S.sintoni*, and *S. sumbarica* respectively.

**Table 4.** The sex ratio of phlebotomine sand flies collected by different traps, North Khorasan Province, 2016

**CM**	***Phlebotomus***				***Sergentomyia***							

	**Female**	**Male**	**Total**	**SR**	**Female**	**Male**	**Total**	**SR**
**ABT**	191	257	448	135	29	31	60	107
**BST**	467	271	738	58	41	6	47	15
**CLT**	856	733	1589	86	33	17	50	52
**DST**	497	613	1110	123	52	41	93	79
**LTP**	424	466	890	110	34	27	61	79
**SPT**	342	850	1192	249	275	147	422	53
**WST**	284	240	524	85	18	11	29	61
**Total**	3061	3430	6491	112	482	280	762	58

CM: Collection methods; ABT: Animal baited trap; BST: Black Shannon trap; CLT: Light trap baited with Carbon dioxide; DST: Disney trap; LTP: CDC Light Trap; SPT: Sticky paper traps; WST: White Shannon trap

Species diversity based on Shannon-Wiener index and species richness has been calculated to estimate species biodiversity of Phlebotomine sand flies in different collection methods in study area and is shown in Table 5.

**Table 5.** Species diversity and species richness of Phlebotomine sand flies collected by different traps, North Khorasan Province, 2016

**Collection methods**	**ABT**	**BST**	**CLT**	**DST**	**LTP**	**SPT**	**WST**
**species richness**	12	11	13	15	16	18	8
**Species diversity based on Shannon-Wiener index**	1.39	1.05	0.99	0.88	1.09	1.60	1.01

ABT: Animal baited trap; BST: Black Shannon trap; CLT: Light trap baited with Carbon dioxide; DST: Disney trap; LTP: CDC Light Trap; SPT: Sticky paper traps; WST: White Shannon trap

## Discussion

This is the first research conducted in northeast of Iran to evaluate different sand fly collection methods in three endemic foci of cutaneous and visceral leishmaniasis in North Khorasan Province, Iran. A total of 19 species of sand flies collected and identified. Some proven or suspected vectors of CL and VL in Iran including *Ph. papatasi*, *Ph. sergenti*, *Ph. caucasicus*, *Ph. alexandri* and *Ph. major* were collected in the study area.

Our findings confirm that these traps differ in performance and demonstrated that CLT and SPT are the most efficient sand fly collection methods. CLT collected 24.5% of all *Phlebotomus* genus, 13 species and its Shannon-Wiener index was 0.99 while SPT collected 18.4% of *Phlebotomus* genus, 55.4% of *Sergentomyia* genus, 18 species of Phlebotominae sand flies and its Shannon-Wiener index was 1.60.

The result of the performance of CLT and SPT is in agreement with some studies about the productivity of these methods (10, 12) but is in contrast with the result of some studies (11, 13). It seems in arid and dry area the performance of SPT is more appropriate than other methods, but in moisture and mountainous area this method has low efficiency. SPT become ineffective in habitats with high relative humidity because of the viscosity of castorl oil and only dead specimens are collected (20). In our study the performance of SPT in arid area was more than mountainous area.

Species with very low abundance may be difficult to detect using non-attractive traps such as SPT. However, we captured some of rare species only with SPT, so this method could assess the species composition and is a perfect method for determination of biodiversity indices of phlebotominae sand flies. Collection of high abundance of *Sergentomyia* genus and male specimens illustrated in this method.

According to our study, CLT seems to be one of the most effective collection methods and could assess the species composition of Phlebotominae sand flies. This trap showed higher performance in capturing females and *Phlebotomus* specimens; therefore CLT can be used for monitoring the population of *Phlebotomus* genus during surveillance. It seems in moisture and mountainous areas this method has high efficiency and the result is in agreement with some studies. Some researchers obtained CO_2_ for CLT using dry ice, which is difficult for regulation of gas emission so we used a CO_2_ gas tank could easily, regulated and increased the efficacy of this method.

The performance of CLT was more than LTP. CO_2_ is usually a long-range attractant for sand flies, whereas light is probably perceived by sand flies at much closer range (20).

## Conclusion

Our results suggest that CLT and SPT are suitable for entomological surveys in the country. CLT is higher attractive for females and *Phlebotomus* genus and is an ideal method for monitoring the population of *Phlebotomus* genus during surveillance. However, SPT is an inexpensive, convenient and easy to be used to detect the presence of sand flies at low densities and captured some of rare species and provide a more realistic estimation of sand flies biodiversity. Therefore SPT is probably the best choice for monitoring of Phlebotominae sand flies.
